# Supplementary material for: Structural dynamics of basaltic melt at mantle conditions with implications for magma oceans and superplumes
Source: Nat Commun. 2020 Sep 23;11:4815. doi: 10.1038/s41467-020-18660-w (PMC7511909; doi:10.1038/s41467-020-18660-w)
Supplement: Supplementary file 1 — Supplementary Information [file 41467_2020_18660_MOESM1_ESM.pdf]

Supplementary Information for

Structural dynamics of basaltic melt at mantle conditions with implications for magma oceans and superplumes

Arnab Majumdar[1], Min Wu[1,2], Yuanming Pan[3], Toshiaki Iitaka[4] and John S. Tse[1*]

[1] Department of Physics and Engineering Physics, University of Saskatchewan, Saskatoon Canada, S7N 5E2, Canada

[2] College of Materials Science and Engineering, Zhejiang University of Technology, Hangzhou, 310014, P. R. China

[3] Department of Geological Sciences, University of Saskatchewan, Saskatoon, S7N 5E2, Canada

[4] Discrete Event Simulation Research Team, RIKEN Center for Computational Science (R-CCS), 2-1 Hirosawa, Wako, Saitama, 351-0198, Japan

[*] john.tse@usask.ca

Supplementary Note 1. Equation of state, density, bulk modulus and bulk velocity of sound of the model basaltic melt at 2200 K

To validate the our theoretical procedure, the calculated equation of states (EOS) and density of the model basalt melt at 2200K are succinctly summarized and compared with previous results in Supplementary Figure 1. In view of the difference in the compositions and computational methods the agreements are satisfactory. In particular, the densities (Supplementary Figure 1b) predicted here are in excellent agreement with the simulations performed by Bajgain *et al.* on molten basalt with a similar composition[1]. The P-V data are fit using the 4th order Birch-Murnaghan EOS fit. From the fit, the parameters obtained at 0 GPa are volume at ambient pressure, V_0 = 3120.52 Å^3, bulk modulus, B_0 = 13.0 GPa, and pressure derivative of the bulk modulus, $B_0' =$ 3.2. The bulk velocity of sound evaluated from the EOS first increases rapidly below 20 GPa but then varies almost linearly with pressure at higher pressures (Supplementary Figure 1d). Interestingly, the predicted bulk sound velocities are very similar to the silica glass obtained from experiments and computer simulations (Supplementary Figure 1d).

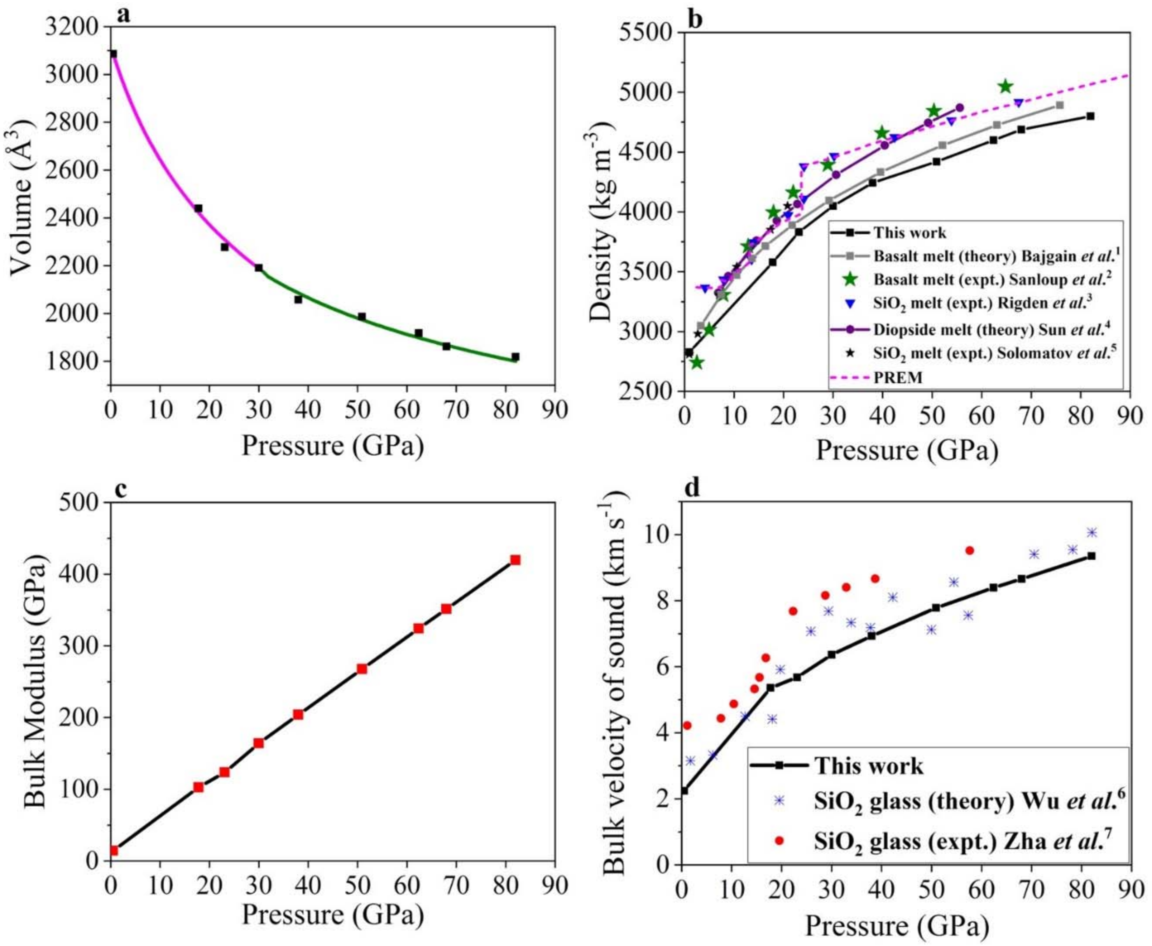

Supplementary Figure 1. Equation of state and mechanical properties. a) P-V curve of the model basaltic melt at 2200 K fit with 4th order Birch- Murnaghan EOS fit. The two different curves are due to two different 4th order BM EOS fits. b) Change in density of the basaltic melt at 2200 K with pressure compared with other silicate melts[1–5]. c) Bulk modulus of the basaltic melt as a function of pressure at 2200 K. d) Pressure evolution of the velocity of sound in the basaltic melt at 2200 K, compared with that of silica glass[6,7].

Supplementary Note 2. Variation of radial distribution functions and average bond lengths

Supplementary Figure 2a shows the radial distribution functions (RDFs) of Si-O in the basaltic melt as a function of pressure at 2200 K. The first peak of the pair distribution function is the nearest Si-O bond length. The Si-O bond length at ambient pressure is 1.62 Å and decreases to 1.60 Å at 18 GPa and then increases gradually above 18 GPa until 50 GPa. But then once again, it decreases with further increase in pressure (Supplementary Figure 2b). The variation of the Al-O

bond length follows an opposite pattern (Supplementary Figure 2c), in which the average bond length increases from 1.75 Å at ambient pressure to 1.77 Å at 18 GPa and then decreases with increase in pressure (Supplementary Figure 2d). However, the Al-O bond length does not change appreciably from 1.75 Å between 68 and 82 GPa. In comparison, the Ca-O and Mg-O distances in the basaltic melt decrease with pressure (Supplementary Figure 3) indicating compaction of the Ca and Mg polyhedra. Closer examination shows that the Ca-O and Mg-O bond lengths at pressures above 23 GPa decrease much faster than those at low pressure. The average Ca-O (Supplementary Figure 3b) and Mg-O (Supplementary Figure 3d) bond lengths at ambient pressure are 2.29 Å and 1.95 Å, respectively, and remain relatively constant up to 23 GPa. The Mg-O bond length is shortened sharply at 23 GPa but attains a nearly constant value of 1.90 Å above 50 GPa. It is noteworthy that the Ca-O and Mg-O bond lengths and their pressure trends in the basaltic melt compare reasonably well with those found for the diopside melt as determined by Sun *et al.*[4]

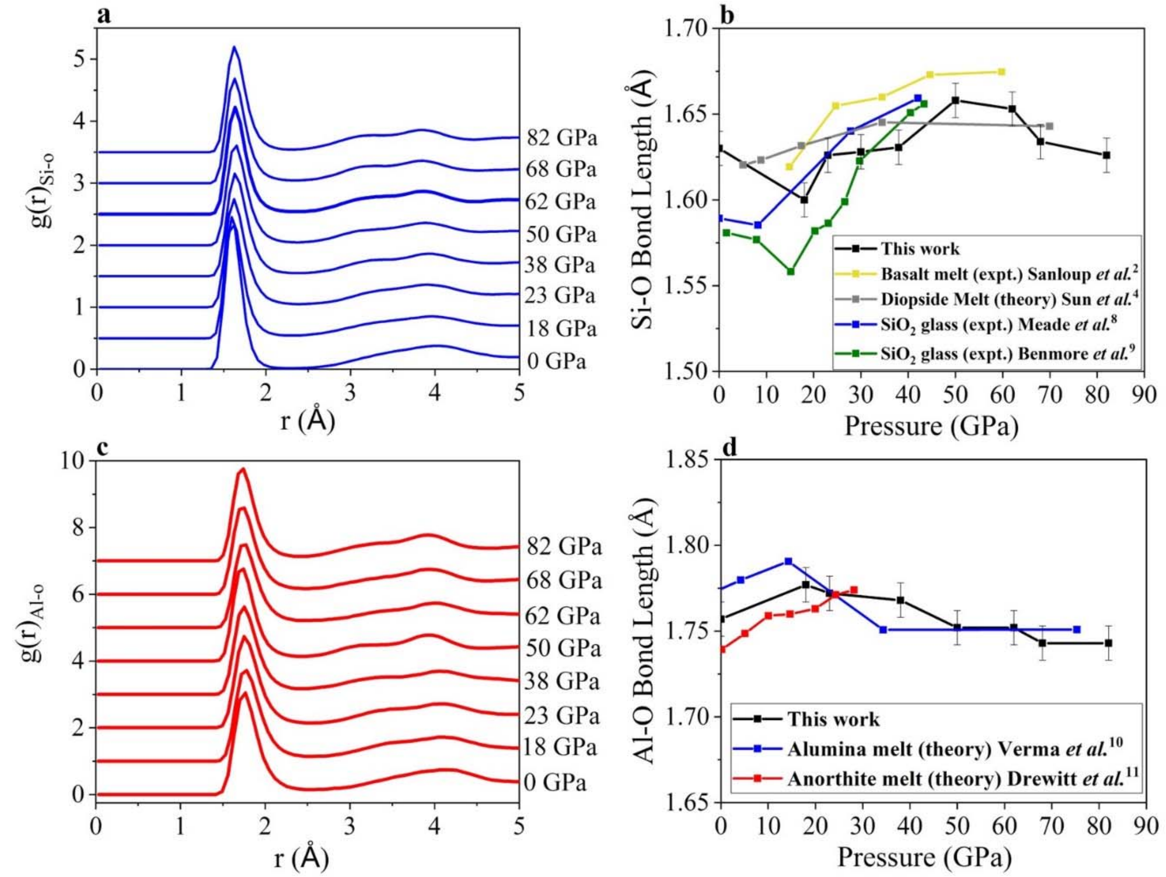

Supplementary Figure 2. Radial distribution function (RDF) and bond lengths. a) The RDF of Si-O in the basaltic melt at 2200 K with increasing pressure and b) Si-O bond length versus pressure, also included for comparison are selected literature data[2,4,8,9]. c) The RDF of Al-O in the basaltic melt at 2200 K at increasing pressure and d) Al-O bond length versus pressure compared with that of alumina[10] and anorthite melt[11]. The error bars pertaining to the mean values of the bond lengths have been determined from the uncertainty of the first peak position of the RDF.

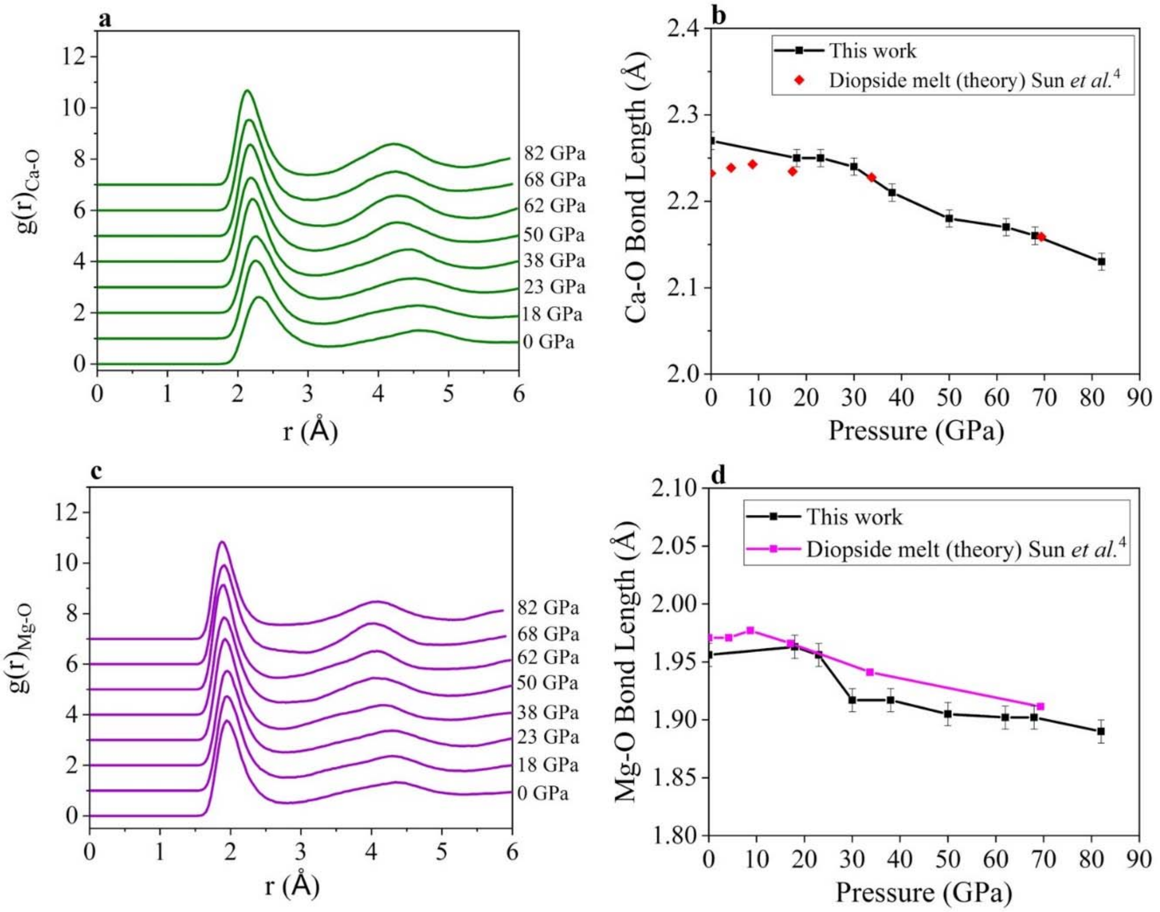

Supplementary Figure 3. Radial distribution function (RDF) and bond lengths. a) The RDF of Ca-O in the basaltic melt at increasing pressure at 2200 K and b) Ca-O bond length versus pressure. c) The RDF of Mg-O in the basaltic melt at increasing pressure at 2200 K and d) Mg-O bond length versus pressure. The error bars pertaining to the mean values of the bond lengths have been determined from the uncertainty of the first peak position of the RDF.

Supplementary Note 3. Bond angle distribution

The bond angle distribution plots for the O-Si-O and O-Al-O for different pressure points at 2200 K have been shown in Supplementary Figure 4. The ~90° is the octahedral O-Si-O and the ~170° is the axial (linear) O-Si-O (Supplementary Figure 4a). It is evident that the local environment is almost octahedral. Compared to the O-Si-O, the O-Al-O becomes 6 coordinated much more rapidly (Supplementary Figure 4b).

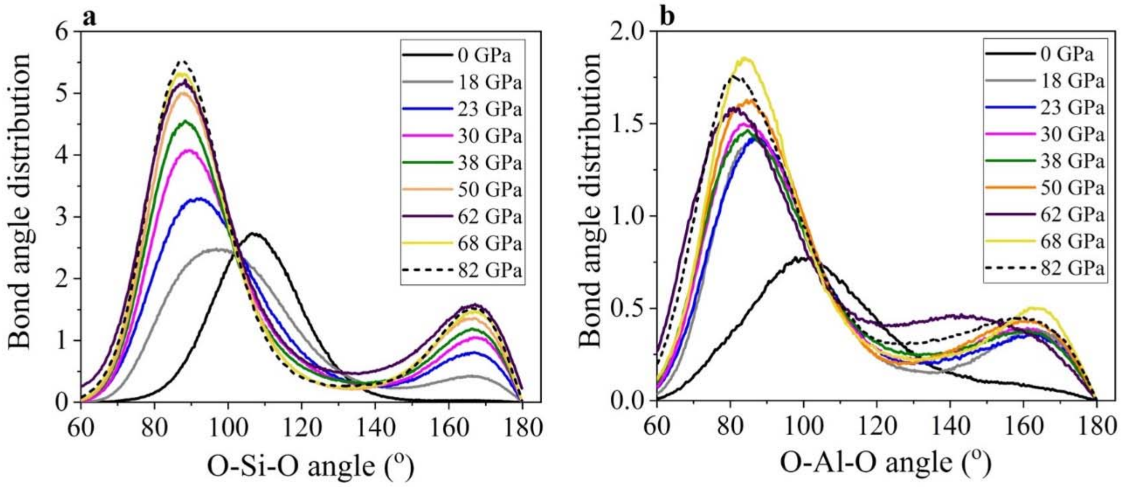

Supplementary Figure 4. Bond angle distribution at 2200 K. a) For O-Si-O and b) O-Al-O angles as a function of pressure.

Supplementary Note 4. Structural transformation of the model basaltic melt with pressure

On compression, both Si-O and Al-O polyhedra show increasing five- and seven-fold coordinations as shown in Supplementary Figures 5 and 6. This has been proposed to be a contributing factor to increasing the non-bridging oxygen and thus increasing the ionic mobility. From Supplementary Figure 5, it can be noted that at 0 GPa, all the Si atoms are four fold coordinated with O atoms. On compression, five-fold coordination can be seen and at 38 GPa, there is a mixture of both five and six-fold coordination. At higher pressures above 50 GPa, mostly six-fold coordination can be seen. A very similar trend is seen for Al-O bonding in Supplementary Figure 6. Although, for the case of Al-O, the four-fold coordination is lost faster and by 18 GPa mostly five-fold coordination can be seen.

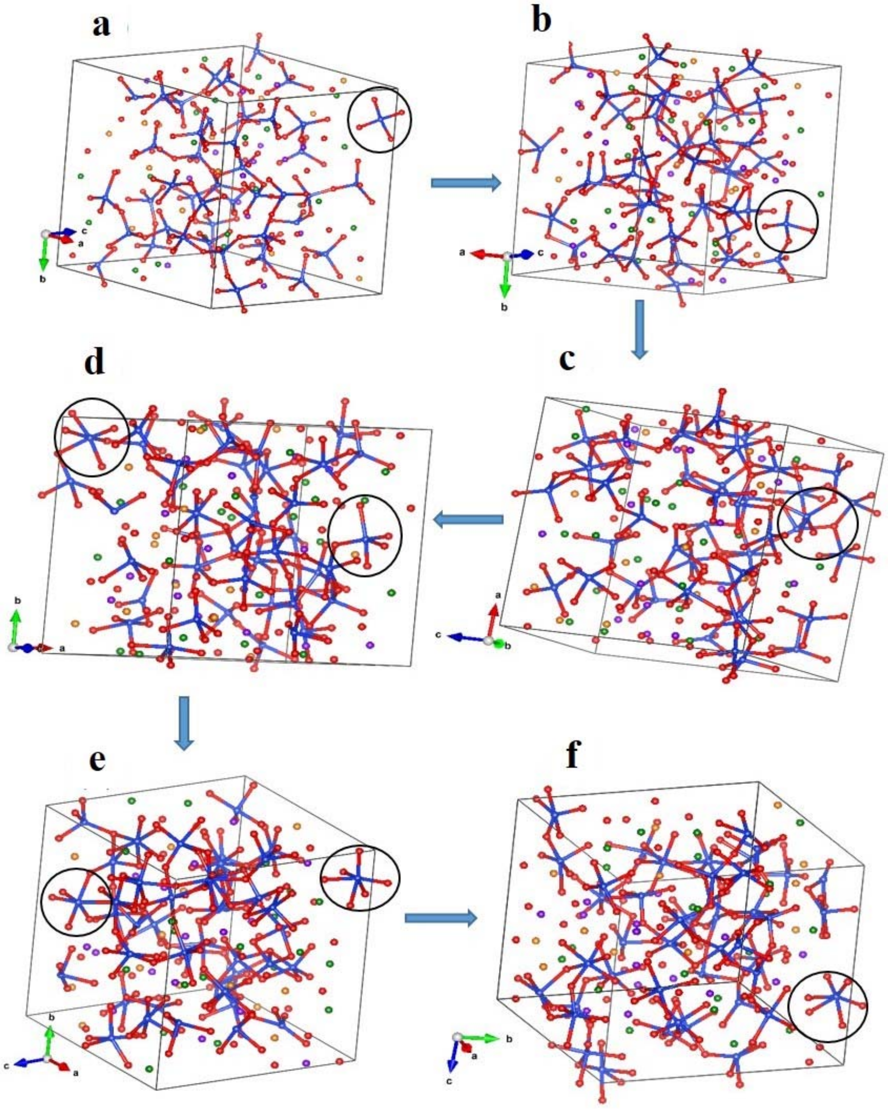

Supplementary Figure 5. Snapshots of the structures of the basaltic melt at 2200 K. At a) 0 GPa, b) 18 GPa, c) 23 GPa, d) 38 GPa, e) 50 GPa, and f) 68 GPa, showing the variation of the Si-O coordination environment with pressure. The blue and red spheres are the Si and O atoms, respectively.

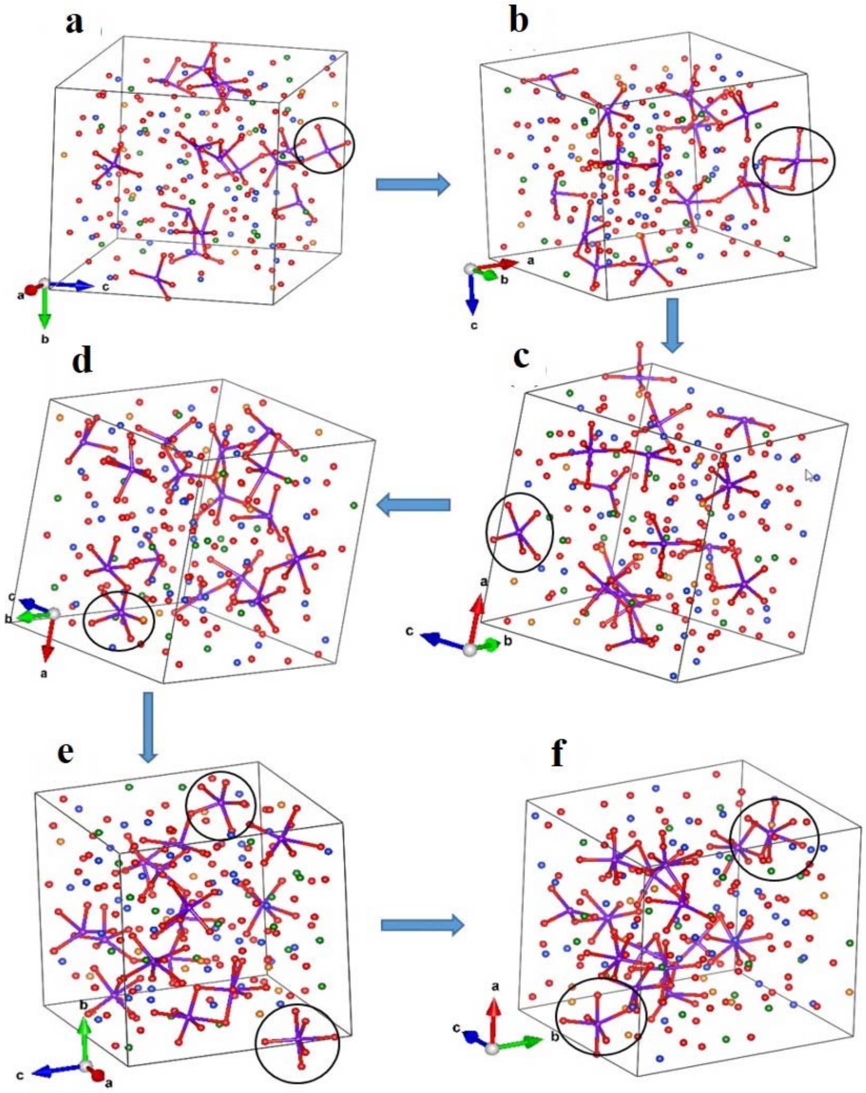

Supplementary Figure 6. Snapshots of the structures of the basaltic melt at 2200 K. At a) 0 GPa, b) 18 GPa, c) 23 GPa, d) 38 GPa, e) 50 GPa, and f) 68 GPa, showing the variation of the Al-O coordination environment with pressure. The purple and red spheres are the Al and O atoms, respectively.

Supplementary Note 5. Coordination number evolution with pressure at 3000 K

The simulations of basaltic melt at 3000 K show a similar structural transformation sequence as that of 2200 K. This has been confirmed by calculating the coordination number of the atomic species at 3000 K between 40-70 GPa and has been shown in Supplementary Figure 7.

However, as expected, due to the increased temperature five- and six-fold coordinated Si-O and Al-O (Supplementary Figure 7) appear at pressures lower than those at 2200 K.

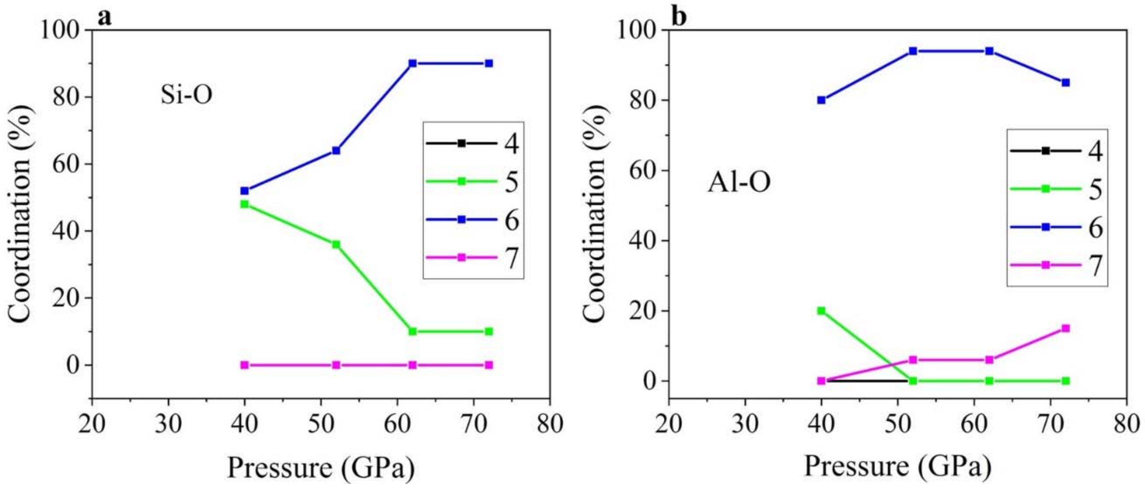

Supplementary Figure 7. Coordination number vs pressure at 3000 K. For the cases of a) Si and b) Al.

Supplementary Note 6. Mean squared displacement and diffusion coefficient of the atomic species

In Supplementary Figure 8, the mean squared displacement (MSD) for the model basaltic melt is shown at 0 GPa and 2200 K. Approximately, after 5 ps, the MSD becomes almost linear. From linear fits, the slope is determined which is employed in the calculation of the diffusion coefficient.

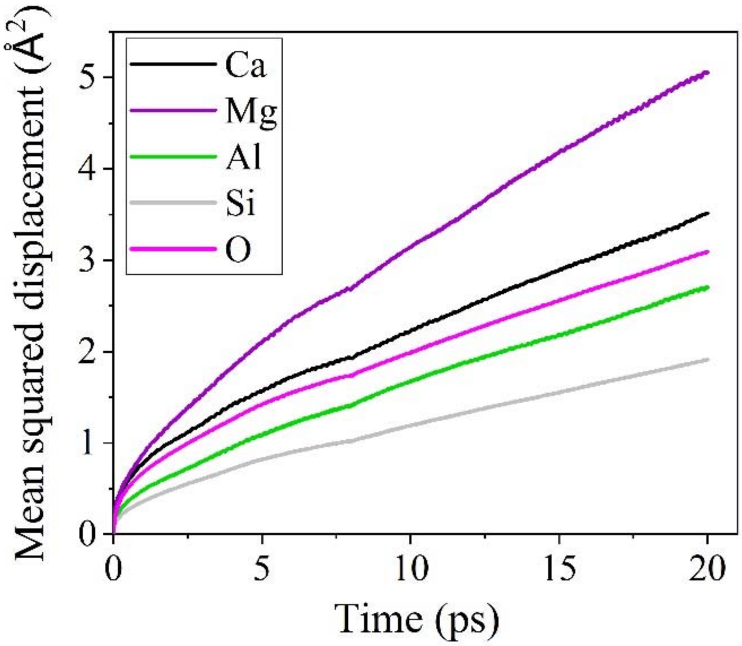

Supplementary Figure 8. Mean squared displacement. The MSD for 0 GPa and 2200 K has been shown.

In Supplementary Figure 9, our calculated diffusion coefficients at 3000 K have been compared with those of similar melts. The order of magnitude matches well, however in our case, we see a sudden increase at 50 GPa after which the diffusion coefficients decrease again. This is consistent with our results obtained for 2200 K for which we see a similar trend.

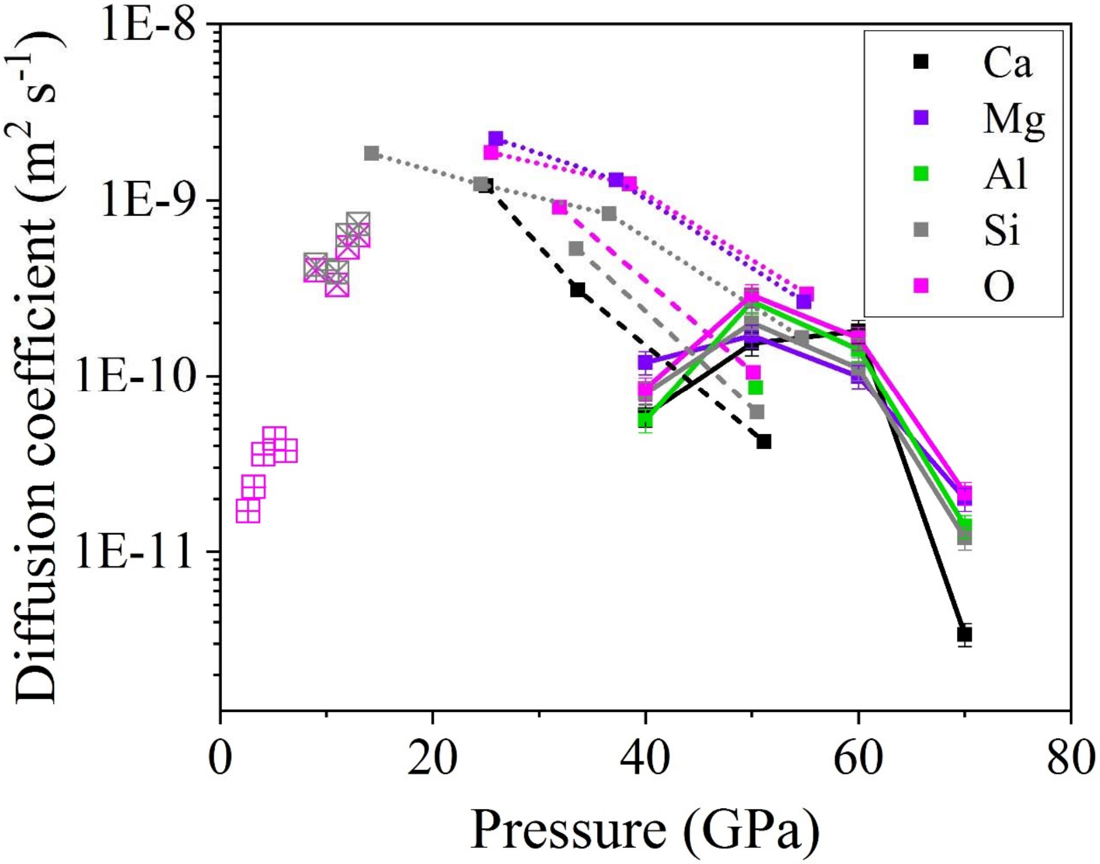

Supplementary Figure 9. Comparison of diffusion coefficients. The solid lines indicate our study at 3000 K. The errors of the mean were determined by considering (i) different intervals of the time origin of the molecular dynamics steps and (ii) minor variation in slope of the straight line used to fit the mean squared displacement. The dashed lines represent the theoretical data obtained for anorthite melt at 3000 K by Karki *et al.*[12] The dotted lines represent the theoretical data obtained for carbonated $MgSiO_3$ by Ghosh and Karki[13]. The squares with 'plus' symbol represent the experimental data obtained by Poe *et al.*[14] for $NaAlSi_3O_8$ at 2100 K. The squares with 'cross' symbol represent the experimental data obtained by Reid e*t al.* for diopside liquid at 2200 K[15].

Supplementary Note 7. Comparison of coefficient of viscosity with other melts

In Supplementary Figure 10, we have compared our calculated coefficient of viscosity with that of other related melts (reported both from experiments and theory), including basaltic magma[16], $MgSiO_3$[17,18], and anorthite[12] and mid-ocean ridge basalt (MORB)[19].

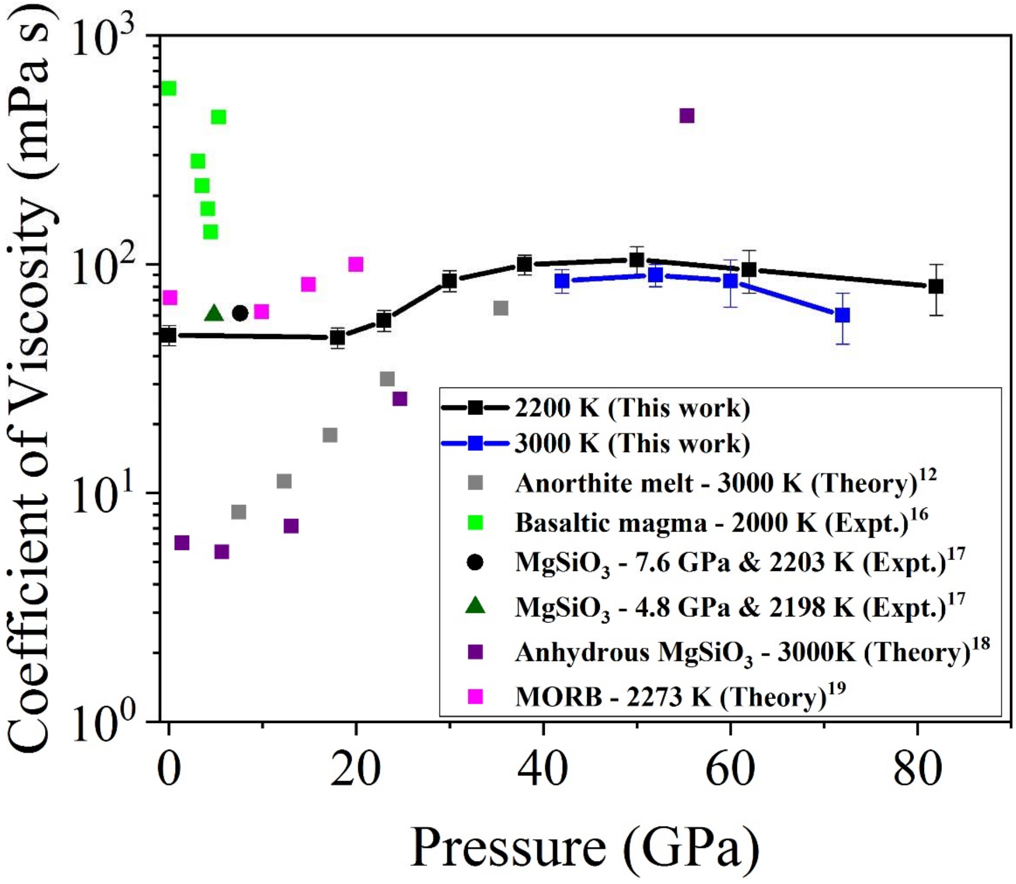

Supplementary Figure 10. Comparison of coeffiecient of viscosity. The standard error of the mean values were determined by considering two possible sources of error in the calculations, which are (i) interval of the time origin of the molecular dynamics steps and (ii) oscillatory nature of the stress auto correlation function after it decays to zero which is integrated to obtain the coefficient of viscosity. Our results have been compared with that of other experimental[16,17] and theoretical[12,18,19] works.

Supplementary Note 8. Comparison of magnesium pseudopotentials

In order to reduce the computation expense significantly, the two-electron pseudopotential has been utilized for Mg instead of the *p*-valence one. The results have been compared in Supplementary Figure 11 for face centered cubic MgO as an exercise and it can be seen that the two pseudopotentials give almost identical results.

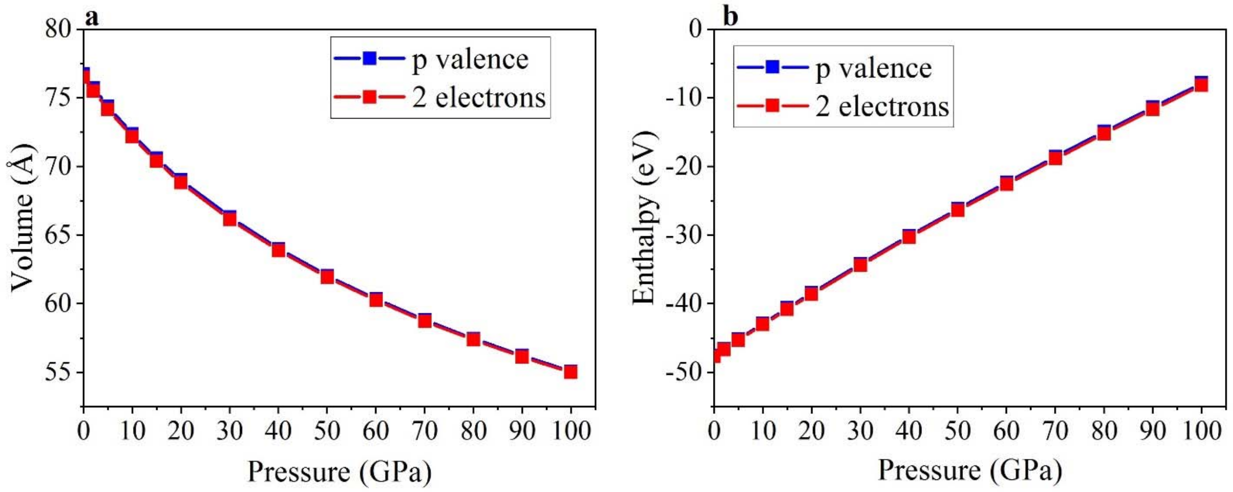

Supplementary Figure 11. **Comparison of two-electron and p-valence pseudopotential. of Mg**. a) P-V curve and b) Enthalpy vs pressure of face centered cubic MgO.

Supplementary Note 9. Convergence of stress autocorrelation function and coefficient of viscosity

The decay of the stress autocorrelation function (SACF) with time has been represented in Supplementary Figure 12 for the cases of 0, 38 and 62 GPa at 2200 K. The SACF decays before ~10 ps and oscillates about zero thereafter. The convergence of the coefficient of viscosity with time has been shown for the cases of 0, 38 and 62 GPa at 2200 K in Supplementary Figure 13. As expected, by ~10 ps, coefficients of viscosity for the different pressure points converge to an average value.

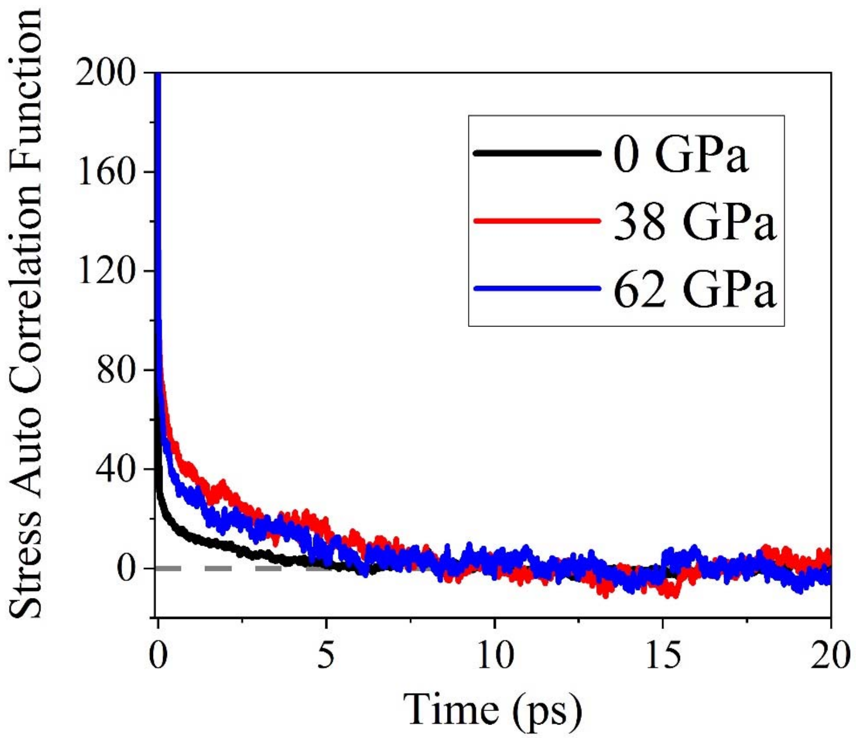

Supplementary Figure 12. Decay of the stress autocorrelation function. The cases for 0, 38 and 62 GPa at 2200 K have been shown.

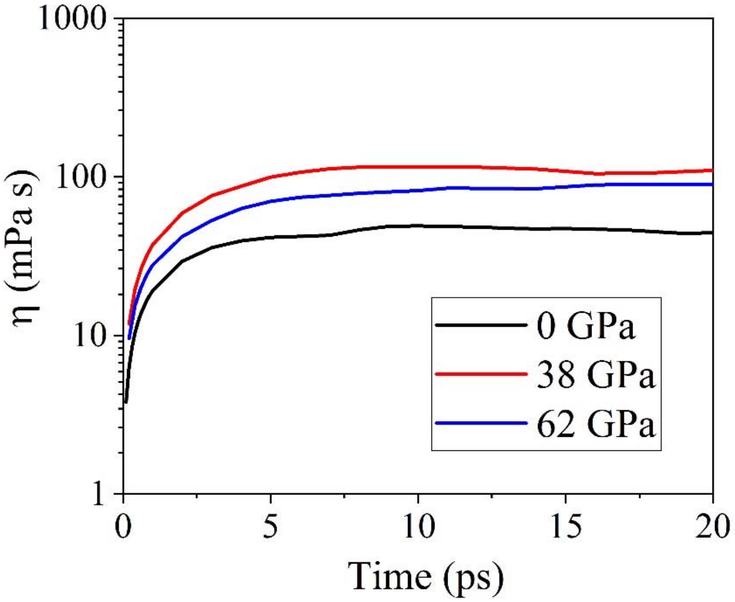

Supplementary Figure 13. Convergence of the coefficient of the viscosity as a function of time. The cases for 0, 38 and 62 GPa at 2200 K have been shown.

Supplementary References

1. Bajgain, S., Ghosh, D. B. & Karki, B. B. Structure and density of basaltic melts at mantle conditions from first-principles simulations. *Nat. Commun.* **6,** 8578 (2015).

2. Sanloup, C. *et al.* Structural change in molten basalt at deep mantle conditions. *Nature* **503,** 104–107 (2013).

3. Rigden, S. M., Ahrens, T. J. & Stolper, E. M. Densities of liquid silicates at high pressures. *Science* **226,** 1071–1075 (1984).

4. Sun, N., Stixrude, L., Koker, N. De & Karki, B. B. First principles molecular dynamics simulations of diopside ($CaMgSi_2O_6$) liquid to high pressure. *Geochim. Cosmochim. Acta* **75,** 3792–3802 (2011).

5. Solomatov, V. *Magma oceans and primordial mantle differentiation*. *Treatise on geophysics* (Elsevier B.V., 2015). doi:10.1016/B978-0-444-53802-4.00155-X

6. Wu, M., Liang, Y., Jiang, J. & Tse, J. S. Structure and properties of dense silica glass. *Sci. Rep.* **2,** 398 (2012).

7. Zha, C., Hemley, R. J., Mao, H., Duffy, T. S. & Meade, C. Acoustic velocities and refractive index of SiO_2 glass to 57.5 GPa by Brillouin scattering. *Phys. Rev. B* **50,** 13105–13112 (1994).

8. Meade, C., Hemley, R. J. & Mao, H. K. High-pressure X-ray diffraction of SiO_2 glass. *Phys. Rev. Lett.* **69,** 1387–1391 (1992).

9. Benmore, C. J. *et al.* Structural and topological changes in silica glass at pressure. *Phys. Rev. B* **81,** 054105 (2010).

10. Verma, A. K., Modak, P. & Karki, B. B. First-principles simulations of thermodynamical and structural properties of liquid Al_2O_3 under pressure. *Phys. Rev. B* **84,** 174116 (2011).

11. Drewitt, J. W. E. *et al.* Development of chemical and topological structure in aluminosilicate liquids and glasses at high pressure. *J. Phys. Condens. Matter* **27,** 105103 (2015).

12. Karki, B. B., Bohara, B. & Stixrude, L. First-principles study of diffusion and viscosity of anorthite ($CaAl_2Si_2O_8$) liquid at high pressure. *Am. Mineral.* **96,** 744–751 (2011).

13. Ghosh, D. B. & Karki, B. B. Transport properties of carbonated silicate melt at high pressure. *Sci. Adv.* **3,** e1701840 (2017).

14. Poe, B. T. *et al.* Silicon and oxygen self-diffusivities in silicate liquids measured to 15

gigapascals and 2800 Kelvin. *Science* **276,** 1245–1248 (1997).

15. Reid, J. E., Poe, B. T., Rubie, D. C., Zotov, N. & Wiedenbeck, M. The self-diffusion of silicon and oxygen in diopside ($CaMgSi_2O_6$) liquid up to 15 GPa. *Chem. Geol.* **174,** 77–86 (2001).

16. Sakamaki, T. *et al.* Ponded melt at the boundary between the lithosphere and asthenosphere. *Nat. Geosci.* **6,** 1041–1044 (2013).

17. Cochain, B., Sanloup, C., Leroy, C. & Kono, Y. Viscosity of mafic magmas at high pressures. *Geophys. Res. Lett.* **44,** 818–826 (2017).

18. Karki, B. B. & Stixrude, L. Viscosity of $MgSiO_3$ liquid at earth's mantle conditions: implications for an early magma ocean. *Science* **328,** 740–742 (2010).

19. Dufils, T., Folliet, N., Mantisi, B., Sator, N. & Guillot, B. Properties of magmatic liquids by molecular dynamics simulation: The example of a MORB melt. *Chem. Geol.* **461,** 34–46 (2017).
